# Perceived barriers of migrants and refugees to vaccinate their children against Measles and polio: a study in Iran

**DOI:** 10.1186/s12939-023-02075-2

**Published:** 2023-12-06

**Authors:** Amir Nasiri, Hossein Farshidi, Farshid Rezaei, Tahereh Dehdari, Afrouzeh Kazemi, Hamid Rezapour, Massomeh Goshtaei

**Affiliations:** 1 Department of Health Education and Health Promotion, School of Public Health, Iran University of Medical Sciences, Tehran, Iran; 2 Rajaie Cardiovascular, Medical and Research Center, Iran University of Medical Sciences, Tehran, Iran; 3 Health Education and Promotion Department, Deputy of Public Health, MOHME, Tehran, Iran; 4 Health Promotion Research Center, Iran University of Medical Sciences, Tehran, Iran; 5 Health Education and Promotion Department, Deputy of Public Health, Iran University of Medical Sciences, Tehran, Iran

**Keywords:** Perceived barriers, Migrant and refugee people, Measles and polio vaccination, Iran

## Abstract

**Background:**

This study examined the perceived barriers of migrants and refugees to vaccinating their children against measles and polio in Iran.

**Methods:**

First, an instrument was developed and validated through several steps. Next, 1,067 parents who had not vaccinated their children against polio and measles or had delayed receiving any dose of these two vaccines until the age of 15 were selected from 16 provinces and completed the instrument. Finally, the data were analyzed.

**Results:**

The results of the explanatory factor analysis showed that the perceived barriers affecting vaccination against polio and measles vaccines were categorized into five factors: low knowledge, negative attitude, communication challenges, lack of participation in vaccination programs, and problems related to migration and refugees. Additionally, the results indicated a significant difference in the mean score of perceived barriers based on participants’ level of education, economic status, and nationality.

**Conclusion:**

The identified barriers may provide a perspective for developing effective efforts in this area. Interventions should focus on parents with low education and poor economic status.

## Background

Global vaccine programs have prioritized the eradication and elimination of two specific diseases: poliomyelitis and measles [1–3]. Despite the extensive efforts of most countries to eliminate these diseases by increasing immunization coverage in children, there are several challenges to achieving this objective [4–6]. One of the challenges is the immunity gap between refugee and migrant children compared to non-migrant children born in host countries [7–9]. Children from migrant families are at a higher risk for certain vaccine-preventable diseases [10].

The increasing public health concern of parental reluctance towards recommended childhood vaccines is influenced by various factors at the individual, vaccine, and environmental levels [11]. Many studies have evaluated the barriers to national program vaccination in refugees and migrants, regardless of the specific age group and vaccine type [12–14]. However, limited studies have documented the personal perceived barriers and perspectives of refugee and migrant parents regarding the uptake of childhood vaccines such as measles and polio. Different reasons have been mentioned for the low coverage of measles or polio vaccination among migrant children. Literature indicates that parents’ negative attitudes towards polio and fear of the vaccine, as well as concerns about the safety and side effects of the measles vaccine, are among the factors contributing to the failure of vaccination programs against these two diseases [15–17]. Hu et al. found that living in a single-child family and having a parent who was unaware of the measles supplementary immunization activity or had low trust in the government-administered measles campaign were reasons for low levels of measles vaccination coverage among migrant children compared to all eligible children in Beijing [18]. In another study, Hu et al. found that several factors influenced the receipt of the first and second doses of measles vaccines among migrant children in East China. These factors included being unaware of the necessity for measles vaccination and its schedule, misunderstanding the side effects of the vaccine, and the child being sick during the recommended vaccination period [19]. Khowaja et al. demonstrated that fear of sterility, lack of faith in the polio vaccine, scepticism about polio supplementary immunization activities, and fear that the vaccine might contain religiously forbidden ingredients were reasons for refusing polio vaccination among Pashtuns in Karachi [20].

Iran has been hosting millions of documented and undocumented refugees for the past four decades [21]. This country is at risk of a polio reemergence as it accommodates approximately 2.5 million Afghan refugees, while neighboring countries Afghanistan and Pakistan continue to experience incidents of wild poliovirus cases [4]. In 2022, 214 cases of measles infection were reported in Iran, half of which were non-Iranians [22]. Despite the fact that Iran’s primary healthcare network provides free access and free-of-charge to a majority of healthcare services, including immunization, for refugees and migrants, including undocumented migrants [21], there remains a significant and unidentified population of migrants and undocumented refugees residing in Iran with low participation in vaccination programs [23]. The evidence indicates that migrant and refugee children have lower immunization rates compared to Iranian-born individuals. The prevalence of partial immunization in non-Iranian children was reported to be six times higher than in Iranian children (11.9% vs. 2%) [9].

Considering the importance of gaining a better understanding of the determinants of parental vaccine hesitancy, vaccine uptake, barriers, and demand issues in migrant and refugee groups in each country [24], the present study was conducted. The objective of the study was to determine the perceived barriers of parents of migrants and refugees to vaccinating their children against measles and polio in Iran.

## Methods

### Data resources and participants

This cross-sectional study was conducted in Iran from October 2022 to April 2023. A total of 1,067 parents of migrant and refugee children were selected from 16 provinces of Iran, including Tehran, Markazi, Ghom, Sistan and Baluchistan, Fars, Khuzestan, Kerman, Alborz, Esfahan, Khorasan Razavi, South Khorasan, Semnan, Bushehr, Hormozghan, and Qazvin. These provinces were chosen as they have large numbers of migrants and refugees, making them particularly relevant to the study. The inclusion criteria were: (a) Immigration from other countries to Iran, (b) Parents whose children under the age of 15 had not yet received measles or polio vaccinations, (c) Parents whose children under the age of 15 had experienced at least a delay in receiving the measles or polio vaccine, and (d) Willingness to participate.

In the present study, accessing samples for random sampling was challenging due to several factors. These factors include the residence of some refugees and migrants in the suburbs and remote areas, as well as their lack of a residence permit and fear of going to healthcare centers. Therefore, an available sampling method was used. This method is often employed when the population of interest is difficult to reach or access. In the study, health workers from each province collaborated with health liaisons for non-Iranian nationals in cities and villages, as well as the SINA and SIB, to identify eligible parents for participation.

It is important to mention that in Iran, the two most commonly used information systems for recording public health services provided to the population are the integrated health record system, known as the “SIB,” and the integrated information record system, known as the “SINA.” The primary objectives of these systems are to facilitate the efficient distribution of health services, establish the necessary requirements for the referral system, evaluate the accuracy of public health data, and ultimately enhance the quality of healthcare services. These systems have been implemented in primary healthcare facilities across Iran and are utilized to record all health-related data collected during the delivery of primary healthcare services to the population. Health information of refugees and migrants is recorded in these systems, just like the health information of Iranian nationals [25–27].

### Variables

#### Outcome variable

The primary outcome variable for this study was the perceived barriers of migrants and refugees to vaccinating their children against measles and polio. These barriers were assessed using a 30-item instrument (developed in the present study) consisting of five subscales. Each subscale included a different number of items: low knowledge (5 items), negative attitude (5 items), communication challenges (5 items), lack of participation in vaccination programs (5 items), and problems related to migration and refugees (5 items).

#### Independent variables

The independent variables included demographic characteristics, including education level (illiterate, < 12th grade, and ≥ 12th grade), sex (male or female), occupation status (employee, self-employed, casual laborer, household duties, and retired), language (Dari, Pashto, Tajik, Uzbek, Arabic, and Ordo), nationality (Afghanistan, Pakistan, Iraq, and others), age (years), length of stay in Iran (years), marital status (single or married), self-reported economic status (weak, moderate, good), and the geographic location of residence (city or town, village, and suburbs). These variables were self-reported using a questionnaire.

### Sample size calculation to assess perceived barriers

The sample size was calculated using the formula (n = Z _1−α/2_ pq_/_d^2^), resulting in 1,067 people. In this formula, a confidence interval of 95%, p = 0.5, and d = 0.03 were considered.

To decrease the chance of missing data, a web-based questionnaire was used (developed in https://porsline.ir) where the option to require an answer to each question was set. Therefore, there was no missing data in the present study. Additionally, the questionnaires were completed by face-to-face interview method for participants.

### Stages of developing the instrument

In the present study, researchers developed an instrument to gather information on perceived barriers of migrants and refugees to vaccinating their children against measles and polio. Firstly, item generation (n = 40 items) was carried out based on a literature review and 15 face-to-face interviews with health liaisons for non-Iranian nationals and five health workers. Next, several tests were conducted to measure the validity and reliability of the developed instrument. The findings of each test are reported in the following section.

#### Quantitative and qualitative content validity

A panel of six experts in public health and infectious diseases evaluated the quantitative and qualitative content validities of the instrument items. They assessed the Content Validity Index (CVI) and Content Validity Ratio (CVR) of each item [28, 29]. The CVR formula is calculated based on the level of agreement among the experts who evaluate an item as essential [28]. The panelists rated the necessity of the items using a three-point rating scale: essential, useful but not essential, and not necessary. The CVI was calculated by dividing the number of experts who assessed an item as essential or very relevant by their total number. Additionally, the relevance of the items was assessed using a four-point rating scale ranging from not relevant to very relevant.

#### Face validity

The developed instrument underwent qualitative and quantitative face validity assessment by ten health liaisons for non-Iranian nationals. They evaluated the relevance, ambiguity, and difficulty of the items. Based on their opinions, minor wording errors were edited. The impact score of each item was measured at this stage, with an impact score of ≥ 1.5 considered acceptable [30].

#### KMO and Bartlett’s test and factor analysis

Exploratory Factor Analysis (EFA) using the orthogonal varimax rotation procedure was conducted for the proposed research model. Four hundred parents of migrant and refugee children who met the inclusion criteria completed the instrument (Table 1). To assess the sample adequacy and the appropriateness of the factor analysis model, we performed the Kaiser-Meyer-Olkin (KMO) test and Bartlett’s test of sphericity using SPSS on the instrument. The final list of items in each subscale included in the proposed research model was selected based on commonality indexes above the threshold of 0.4. Additionally, a latent root criterion of 1.0 was used for factor extraction [24, 25].

#### Convergent validity

Based on the data of 400 parents in the EFA assessment stage, the convergent validity of the subscales was measured by Average Variance Extracted (AVE). To establish convergent validity, the constructs’ AVE should exceed 0.50 and be less than Composite Reliability (CR) [31]. The convergent validity of the instrument was measured using Excel software.

#### Cronbach’s alpha

To assess the internal consistency of the instrument’s five subscales, we used Cronbach’s alpha based on the data of 100 parents of migrant and refugee children. An estimate of Cronbach’s alpha (≥ 0.70) was considered satisfactory [32].

### Statistical analyses

The Kolmogorov-Smirnov test was used to verify the normality of the data. Multiple linear regression (forward method) was used to examine the relationship between perceived barriers and demographic variables in the participants. Post-hoc comparisons were performed using Tukey’s honestly significant difference (HSD) test among various groups based on occupation status, education level, economic status, nationality, and language. The participants’ general characteristics were analyzed using descriptive statistics such as frequency, percentage, mean, and standard deviation. The statistical analysis was conducted using SPSS version 13.0, and a significance level of P < 0.05 was considered statistically significant.

## Results

### The results of the validation of the instrument

#### Quantitative and qualitative content validity

After calculating CVI and CVR, eleven items were eliminated at this stage because they had a CVI score of less than 0.79 and a CVR score of less than 0.99. Additionally, two items were edited based on suggestions from an expert panel to clarify ambiguity in their wording (qualitative content analysis).

#### Face validity

After measuring quantitative face validity, four items that did not have an impact score of ≥ 1.5 were deleted.

#### KMO and Bartlett’s test and factor analysis

Table 1 presents the demographic characteristics of the participants in EFA. In this study, the KMO was 0.871, and the Bartlett’s test of sphericity was significant (= 3615.445, df = 562, p < 0.0001), indicating that the data were suitable for factor analysis. Eigenvalues of all subscales were > 1, confirming the suitability of the data for factor analysis [31, 33]. Table 2 displays the factor load of each item. At this stage, one item was deleted. EFA revealed that 25 items could be categorized into five factors: low knowledge, negative attitude, communication challenges, low participation in vaccination programs, and problems related to migration to another country.

**Table 1:** Demographic information of the participants

	Exploratory factor analysis and convergent validity (n = 400)		Perceived barriers (n = 1067)	
	*n*	%	*n*	%
**Sex**				
Woman	337	84.3	785	73.6
Man	63	15.8	282	26.4
**Education level**				
Illiterate	160	40	500	46.9
< 12th grade	233	58.2	477	44.7
≥ 12th grade	7	1.8	90	8.4
**Occupation status**				
Employee	3	0.8	54	5.1
Self-employed	28	7	82	7.7
Causal labourer	74	18.5	244	22.9
Household duties	274	68.4	608	57.0
Retired	21	5.3	79	7.4
**Language**				
Dari	203	50.7	443	41.5
Pashto	63	15.7	229	21.5
Tajik	30	7.5	71	6.7
Uzbek	27	6.8	87	8.2
Arabic	0	0	7	0.7
Ordo	77	19.3	230	21.6
**Nationality**				
Afghanistan	390	97.5	963	90.3
Pakistan	2	0.5	38	3.6
Iraq	2	0.5	6	0.6
Other	6	1.5	60	5.6
**Self-reported economic status**				
Weak	196	49	549	51.5
Moderate	184	46	458	42.9
Good	20	5	60	5.6
**The geographic location of residence**				
City or town	226	56.4	535	50.1
Village	83	20.8	361	33.8
Suburbs	91	22.8	171	16.0

**Table 2:** The results of explanatory factor analysis (n = 400), Cronbach’s alpha (n = 100), ICC (n = 30) and AVE and AR (n = 400)

	Component	Extraction	Cronbach’s alpha	AVE	CR
**Low knowledge**			0.843	0.604	0.968
1. Polio and measles vaccines cannot prevent two measles and poliomyelitis diseases in children.	0.804	0.646			
2. Polio and measles vaccines have no benefit for children.	0.854	0.730			
3. Polio and measles vaccines are harmful for children.	0.810	0.656			
4. It is not necessary for children who were born in Iran and have already received all their vaccines to receive supplementary polio or measles vaccines.	0.728	0.530			
5. Before migrating to Iran, my child had already been vaccinated against polio and measles. I don’t think it is necessary for them receive the vaccinations again in Iran.	0.678	0.460			
**Negative attitude**			0.872	0.556	0.861
7. Migrants who enter a country illegally may be afraid to go to health centers to have their children vaccinated against polio or measles.	0.616	0.379			
8. The aim of polio and measles vaccination is to test the vaccines on the children of the migrants.	0.804	0.646			
9. Polio and measles vaccination aim for the children of the migrants is gene change of the migrants.	0.768	0.618			
10. When polio or measles vaccines are given only to migrants children and not to Iranian children, it is suspicious.	0.762	0.581			
11. As children, we did not receive vaccinations for polio or measles and never became sick. Therefore, why should our children receive vaccinations?	0.745	0.555			
**Low participation in vaccination programs**			0.762	0.516	0.840
12. I don’t know the health liaison for my place of residence.	0.770	0.593			
13. I don’t know how to provide my opinions and suggestions to health officials and providers to increase the coverage of polio and measles vaccination in the children of migrants and refugees.	0.784	0.615			
14. So far, I have not received any counseling or training on the importance of vaccinating children against polio and measles.	0.775	0.601			
15. So far, I have not been invited to participate in the implementation of polio and measles vaccination programs for children of migrants and refugees.	0.671	0.450			
16. The lack of educational programs in our mother tongue and languages (Dari, Uzbek, Pashto, Tajik, Arabic, Urdu, etc.) causes me to be indifferent towards polio and measles vaccination programs.	0.568	0.322			
**Communication challenges**			0.718	0.501	0.832
17. I have heard from others that after vaccination for polio, some children become paralyzed.	0.782	0.611			
18. People around me, considering their negative experiences, have asked me not to do polio and measles vaccination for my children.	0.695	0.484			
19. Migrants who have recently arrived in Iran do not trust the recommendations of Iranian health providers to vaccinate their children against polio or measles.	0.696	0.484			
20. Living on the outskirts of cities and remote areas can hinder or delay the vaccination of my children against polio and measles.	0.647	0.418			
21. My spouse or mother-in-law does not allow me to leave the house to get my child vaccinated.	0.706	0.498			
**Problems related to migration and refugee**			0.700	0.546	0.856
22. My place of residence and my family’s address in Iran frequently change.	0.646	0.418			
23. I don’t have my child’s previous vaccination card, and I don’t know which one of my children has not received their vaccines.	0.772	0.596			
24. The busyness of life and migration prevent me from timely taking my child for vaccination.	0.683	0.467			
25. Due to my unfamiliarity with Iran, I cannot remember my home address accurately and record it in the health file. As a result, healthcare workers cannot find our house for my child’s vaccination.	0.785	0.616			
26. My child does not have a birth certificate, and I do not know their age.	0.798	0.637			

#### Convergent validity

The convergent validity of all subscales was considered acceptable (CR > 0.60 and AVE > 0.50) [31], confirming convergent validity. The AVE and CR of all subscales are reported in Table 2.

#### Cronbach’s alpha

In this study, Cronbach’s alpha of the developed instrument subscales was the range of 0.70–0.843 (Table 2).

#### Final instrument and scaling

The final 25-item instrument consists of five subscales, with 5 items each: low knowledge, negative attitude, communication challenges, lack of participation in vaccination programs, and problems related to migration and refugees. Table 3 displays the statements used to measure each variable. All items were scored on a Likert scale ranging from 1 (completely disagree) to 5 (completely agree).

### The results of measuring perceived barriers and their subscales

#### Demographic characteristics of the participants

The participants had a mean age of 31.84 (SD = 9.391) years old and had lived in Iran for an average of 12.74 (SD = 11.463) years. Table 1 displays other demographic characteristics of the participants.

The results of the simple regression analysis indicated that all demographic variables, except for the age of the parents, were significant at a level of 0.2, making them candidates for multiple linear regression. The findings of the multiple linear regression (forward method) revealed that education level, nationality, and self-reported economic status variables were significant predictors of perceived barriers to vaccination of children against polio and measles (R^2^ = 0.060, F = 9.579, p < 0.001) (Table 3). Increasing one unit in the education level and self-reported economic status variable led to a decrease in perceived barriers to vaccination of children against polio and measles by 0.101 and 0.179, respectively (Table 3). The results of the HSD test indicated that illiterate participants had higher perceived barriers compared to other participants. According to the HSD test results, participants with a good economic status had fewer overall perceived barriers to measles/polio vaccination for their children than participants with poor (p < 0.001) and moderate (p = 0.014) economic status. Refugees and migrants from other nationalities had more perceived barriers to measles/polio vaccination for their children than migrants and refugees from Afghanistan (p < 0.001) and Pakistan (p = 0.001).

**Table 3:** Multiple linear regression model results to investigate demographic factors affecting perceived barriers to vaccination of children against polio and measles (n = 1067)

Variables	Beta	SD	t	p.value
Constant	-	3.876	19.987	< 0.001*
Occupation status	-0.018	0.449	-0.514	0.608
Education level	-0.101	0.417	-3.011	0.003*
Language	-0.044	0.279	-1.380	0.168
Nationality	-0.094	0.843	-2.640	0.008*
Self-reported economic status	-0.179	0.631	-5.569	< 0.001*
The geographic location of residence	0.008	0.688	0.265	0.791
Sex	-0.033	1.279	-0.981	0.327
The length of residence in Iran	-0.040	0.046	-1.294	0.196

P*<0.05 significant

#### Perceived barriers of migrant and refugee parents to vaccinate their children against Measles and polio

The mean score of perceived barriers and its five subscales is presented in Table 4.

**Table 4:** The mean score of perceived barriers to vaccination of children against polio and measles and its 5 subscales (n = 1,067)

Variables	Mean	SD	Minimum score	Maximum score
Low knowledge	11.801	4.496	5	25
Negative attitude	12.835	4.326	5	24
Low participation in vaccination programs	12.454	4.410	5	25
Communication challenges	12.079	4.164	5	25
Problems related to migration	13.171	4.415	5	25
Total score of perceived barriers	62.361	16.942	25	119

## Discussion

The findings of the current study indicate that a lack of awareness regarding polio and measles vaccines is a hindrance to the timely vaccination or non-vaccination of measles and polio among refugee and migrant children in Iran. This observation aligns with the findings of Shafique et al., who found that insufficient knowledge about polio vaccination among individuals was a key factor contributing to the ineffectiveness of Pakistan’s polio eradication program [15]. The significance of immunization knowledge among refugees and migrants was underscored in two studies conducted by Hussain et al. and Abdi et al. [23, 34]. In contrast to our findings, Mishra et al. demonstrated that the awareness of polio vaccination among a sample of Indian mothers was 100%, whereas knowledge about measles vaccination was reported at 83% [35]. Habib et al. demonstrated that there was a considerable level of awareness regarding polio and its immunization among females in Pakistan [36]. From the findings of the studies, it can be concluded that parents of migrant and refugee children possess lower knowledge regarding measles and polio vaccines compared to non-migrant parents in each country. This may be attributed to factors such as lower literacy, language and communication barriers, and limited access to training opportunities in migrants and refugees [8]. As such, the health system in Iran should prioritize raising awareness and knowledge of polio and measles vaccinations among parents of refugees and migrants through targeted campaigns and educational initiatives.

An unfavorable attitude towards polio and measles vaccines was identified as the second barrier that may influence vaccine uptake for children. The findings of Habib et al. were consistent with the results of the present study. According to their report, misperceptions surrounding the polio vaccine resulted in the rejection of both polio vaccines and routine immunizations among females in Pakistan [36]. Shafique et al. found that individuals’ unfavorable attitude towards polio vaccination was one of the main reasons for the failure of Pakistan’s polio eradication program [13]. In another study, Singh and Chawla found that the attitude regarding the measles vaccine was not favorable in women with children under the age of five in an urban slum area of Aligarh [37].

Contrary to our findings, Hussain et al. identified that 96.85% of their participants endorsed the necessity of immunizing children. Despite parents’ insufficient knowledge regarding their children’s immunization, their attitudes towards it remained positive, as highlighted in their study [34]. In another study, Mollema et al. reported that most parents in the Netherlands had a positive attitude towards childhood vaccination, although some had doubts [38]. The reason for the contradiction between the findings of past studies in the field of attitudes towards measles and polio vaccines may be attributed to differences in the target population and geographical area where these studies were conducted. It is essential to conduct periodic needs assessments to identify the requirements of all population groups, such as refugees and migrants, in each society and design suitable interventions accordingly. It is recommended to implement collaborative educational programs, such as peer education, to enhance the positive attitude of refugee and migrant parents towards measles and polio vaccines in Iran.

Communication challenges and low parental participation in vaccination programs have been identified as two primary barriers that can influence the uptake of polio and measles vaccines for children. Common rumors about the possibility of paralysis in children and other negative consequences after receiving the polio and measles vaccines, a lack of trust in the recommendations of Iranian health providers, and living in the outskirts of cities and remote areas have been recognized as challenges that contribute to the delay or non-vaccination of children against measles and polio among the study participants. To the best of our knowledge, most previous studies have identified communication and participation factors as influential factors in the vaccination rate of polio and measles in children. For example, SteelFisher et al. found that increasing trust in vaccinators, providing accurate information about poliovirus transmission, spreading positive messages to counter rumors, and fostering community support for polio vaccination could potentially strengthen caregivers’ commitment to polio vaccinations in Afghanistan [39]. The findings of a study conducted in Sudan indicated that exposure to anti-vaccination information messages or materials and doubts about the effectiveness of the measles vaccine were two significant factors contributing to parental hesitancy regarding the vaccine. The study also concluded that investing in vaccines and addressing accessibility issues could serve as effective interventions for enhancing measles vaccine acceptance and, consequently, improving measles vaccine coverage [16]. Kashyap et al. found that the parent-provider relationship, weak interpersonal communication skills of health workers, social media, and lack of trust may influence parents’ reluctance to vaccinate their children against infectious diseases [40].

In the present study, a small number of participants reported that they had not been invited to participate in the implementation of polio and measles vaccination programs for children of migrants and refugees. While community involvement has a positive effect on health, especially when supported by robust organizational and community procedures [41], consistent with our findings, Itimi et al. showed that childhood immunization coverage was attributed to improved mobilization and participation in the delivery of immunization services [42]. In Iran’s health system, non-Iranian volunteers participate as health liaisons for non-Iranian nationals. They transmit information and follow up on cases of vaccination delays among migrants and refugees in all parts of the country, including suburbs and remote areas. Since some non-Iranian migrants and refugees place more trust in these contacts due to shared language and culture, the health system should take steps to empower these individuals on childhood vaccination and address rumors regarding the vaccine. This empowerment will strengthen their role as intermediaries between the health system and migrants and refugees. It is necessary to provide training for this group on the positive effects of community participation on childhood vaccination coverage, as well as strategies for increasing it.

One of the barriers influencing polio and measles vaccination rates was the problems related to migration. Some of the reported challenges faced by participants included regular changes in the geographical area of residence, unfamiliarity with Iran, lack of birth certificates, and absence of the child’s previous vaccination card. These problems have been reported in previous studies. For example, Assi et al. and Azizi et al. found that the absence of fixed addresses among refugees made it more challenging to deliver primary and secondary healthcare services to them [43,14]. In another study, Hu et al. found that immigration status had an effect on polio and measles vaccination coverage in Zhejiang province, China [44]. The literature has shown that migrants and refugees are a hard-to-reach population for vaccination due to various factors, including geographical distance, transient lifestyles, discrimination by healthcare providers, insufficient vaccination systems, conflicts, and legal restrictions. These barriers limit their access to healthcare and make it challenging to track their vaccination status, leading to missed opportunities for vaccination [45]. When planning to increase vaccination coverage in migrant and refugee children, special attention should be paid to immigration-related problems such as not having a vaccination card, frequent changes of residence, unfamiliarity with the destination country, and more. It is crucial to integrate refugees and migrants into each country’s health system by incorporating them into immunization policies, planning, and service delivery.

The findings showed that there were significant difference between the mean score of perceived barriers according to the participants’ level of education and economic status. Previous studies have consistently identified an association between socioeconomic status and the acceptance of polio and measles vaccination. For example, Shafique et al. found that participants with higher education and better financial status had a greater knowledge about polio vaccination [15]. This finding has been confirmed by the results of studies conducted by Alagsam [46], Kantner et al. [47], Hu et al. [44], and Hossain et al. [48]. To achieve full coverage of measles and polio vaccines among refugees and migrants in Iran, it is crucial to prioritize parents with low education or income levels. Specific vaccination strategies should be implemented to enhance access to these communities.

This study represents the first national-level survey conducted on barriers to vaccinating children against polio and measles among a sample of migrants and refugees in Iran. Despite this, the study has limitations. One limitation is the lack of access to all sub-groups of migrants and refugees, particularly those residing in remote and inaccessible areas or those without permission to stay in Iran. Additionally, there may be subgroups that frequently change their addresses. As a result, Iran’s health system faces additional challenges in reaching these populations. Therefore, it is important to note that these findings may not be generalizable to the entire population of refugees and migrants in Iran. Another limitation of the present study was the inability to evaluate the test-retest reliability of the questionnaire due to problems accessing the participants and changes in their residence between the two visits required to complete the questionnaire. Another limitation of the study is its focus solely on examining the personal perspectives of parents regarding their barriers to child vaccination. Future studies should consider investigating the role of other factors, such as structural factors, in relation to measles and polio vaccination coverage among children of refugees and migrants. Overall, these limitations highlight the need for further research and a comprehensive approach to address the barriers to vaccination among refugees and migrants in Iran.

## Conclusion

This study contributed to a better understanding of the barriers to delayed or non-vaccination of measles and polio for children of refugees and migrants in Iran. The findings of this study suggest that, despite free access and no charge for immunization of children of refugees and migrants, low knowledge, negative attitudes, low participation in vaccination programs, communication challenges, and problems related to migration are recognized as barriers to vaccinating children of refugees and migrants against polio and measles in Iran. These results can provide insights for researchers and policymakers at various levels in developing tailored and targeted programs for caregivers of refugee and migrant children, taking into consideration the barriers to vaccinating children as a whole.

## Data Availability

The datasets used and/or analyzed during the current study are available from the corresponding author upon reasonable request. Please contact the corresponding author for the data requests.

## Abbreviations

CVI: Content Validity Index
CVR: Content Validity Ratio
KMO: Kaiser-Meyer-Olkin test
EFA: Explanatory Factor Analysis
